# Timing of surgical intervention for compartment syndrome in different body region: systematic review of the literature

**DOI:** 10.1186/s13017-020-00339-8

**Published:** 2020-10-21

**Authors:** Federico Coccolini, Mario Improta, Edoardo Picetti, Luigi Branca Vergano, Fausto Catena, Nicola de ’Angelis, Andrea Bertolucci, Andrew W. Kirkpatrick, Massimo Sartelli, Paola Fugazzola, Dario Tartaglia, Massimo Chiarugi

**Affiliations:** 1 General, Emergency and Trauma Surgery Department, Pisa University Hospital, Via Paradisia 1, 56100 Pisa, Italy; 2 General, Emergency and Trauma Surgery Department, Bufalini Hospital, Cesena, Italy; 3 Department of Anesthesia and Intensive Care, Parma University Hospital, Parma, Italy; 4 Orthopedic Department, Bufalini Hospital, Cesena, Italy; 5 Emergency Surgery Department, Parma University Hospital, Parma, Italy; 6 Unit of Digestive and Hepato-biliary-pancreatic Surgery, Henri Mondor Hospital and University Paris-Est Créteil (UPEC), Créteil, France; 7 Departments of Surgery and Critical Care Medicine, Foothills Medical Centre, Calgary, Canada; 8 General Surgery Department, Macerata Hospital, Macerata, Italy; 9 General, Emergency and Trauma Surgery Department, Bufalini Hospital, Cesena, Italy

**Keywords:** Hypertension, Decompressive craniectomy, Compartment syndrome, Extremities, Ocular, Plycompartment

## Abstract

Compartment syndrome can occur in many body regions and may range from homeostasis asymptomatic alterations to severe, life-threatening conditions. Surgical intervention to decompress affected organs or area of the body is often the only effective treatment, although evidences to assess the best timing of intervention are lacking. Present paper systematically reviewed the literature stratifying timings according to the compartmental syndromes which may beneficiate from immediate, early, delayed, or prophylactic surgical decompression. Timing of decompression have been stratified into four categories: (1) *immediate decompression* for those compartmental syndromes whose missed therapy would rapidly lead to patient death or extreme disability, (2) *early decompression* with the time burden of 3–12 h and in any case before clinical signs of irreversible deterioration, (3) *delayed decompression* identified with decompression performed after 12 h or after signs of clinical deterioration has occurred, and (4) *prophylactic decompression* in those situations where high incidence of compartment syndrome is expected after a specific causative event.

## Introduction

The ability to tolerate rise in pressure of a closed area of the body, depends on three main factors: the compliance—the extent to which that region can expand to adjust the increasing pressure—the degree of vascular and nervous damage that occurs in the area, and the physiological effects that the increasing pressure generate on the body homeostasis. It is explicit that some compartments can only briefly tolerate an acute rise in pressure due to its detrimental effect like hemodynamic instability seen in tension pneumothorax or cardiac tamponade even if those pathological entities seldom are included among “compartmental syndromes”. In fact, compartment syndrome properly defined is the pressure increase inside a defined body compartment. When tissue interstitial pressure becomes higher than the capillary one, cells get insufficient blood supply. To revert those scenarios, generally urgent intervention may be required. Uncertainty exists in regard to the timing of intervention of more complex, urgent compartmental syndromes. The resistance of the different tissues to hypoxia and hypoperfusion is unknown. Different tissues, in fact, have different capability to resist to blood flow reduction due to pressure increase. Several compartments exist in the human body. Most of them may potentially develop compartment syndrome but only a few of them may impact on the body homeostasis or may be surgically decompressed. Moreover, only a small part of these areas has been investigated in terms of compartment syndrome effects and management. Present paper included among compartment syndromes even those clinical conditions characterized by a pressure increase impairing the function even if not directly reducing the blood supply to tissues. In fact, tension pneumothorax and cardiac tamponade, with a sudden increase of pressure inside un-expandable districts, may determine life-threatening conditions that must be solved as soon as possible through surgical decompression. The aim of this paper is to evaluate the current evidence in order to assess the optimal timing for surgical intervention in these main compartments. The deep understanding of pathophysiology underlining at the development of each compartment syndrome is beyond the proposal of this paper and will not be addressed.

## Material and methods

**Fig. 1:**
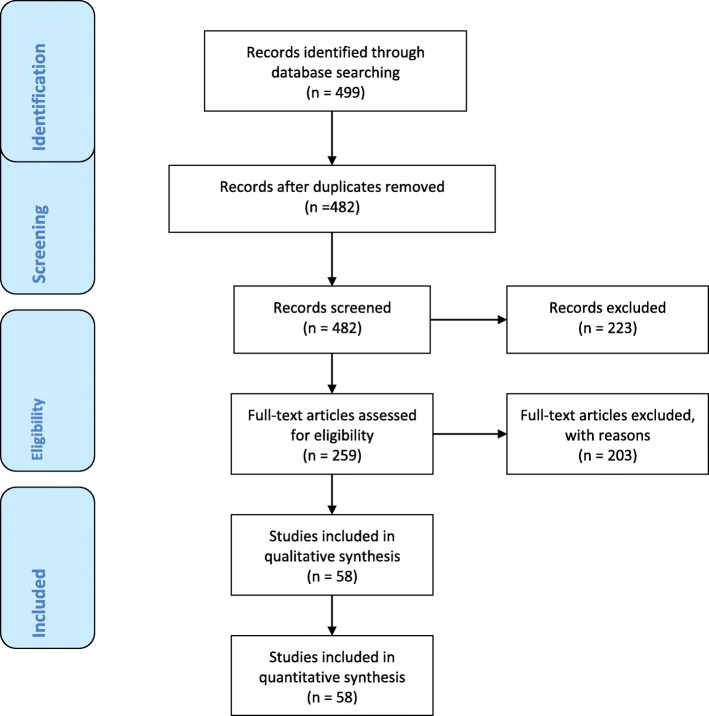
PRISMA flow chart

A systematic computerized search was done in different databanks (MEDLINE, Scopus, EMBASE) citations were included for the period between January 1981 and April 2020 for articles regarding the timing of decompression in compartment syndrome in all body regions. Primary search strategy utilized the following words: abdominal, limb*, ocular, orbital, thora*, hand, compartment, compartmental, compartment* syndr*, lower-limb, upper-limb*, decompressive, decompression, laparotomy, thoracotomy, fasciotomy, acute, vascular, cardiac, non-operative, medical, management, timing, surgery, craniotomy, craniectomy, brain, and cerebral combined with AND/OR. No search restrictions were imposed. The dates were selected to allow comprehensive published abstracts of clinical trials, consensus conference, comparative studies, congresses, guidelines, government publication, multicenter studies, systematic reviews, meta-analysis, large case series, original articles, and randomized controlled trials. Narrative review articles were also analyzed to determine other possible studies. Included articles were enlisted in the tables and selection process is explained in the PRISMA flow chart (Fig. 1).

## Results

A total of 499 articles were retrieved. Among them 58 articles were considered in results analysis (Fig. 1). Included articles are reported in Tables 1, 2, and 3 divided by anatomical regions. Extensive discussion of the results is reported below. Decompression timing was stratified according to priority (Table 4).

**Table 1:** Summary of studies about decompressive procedures in orbital compartment syndrome (residual visual acuity has been uniformed whenever requested from Snellen Imperial to Snellen metric according to Elliott and Flanagan [1])

Author	Year	Number of patients	Disease	Timing	Residual visual acuity	Blindness	Eye loss
Goodall [2]	1999	6	Trauma	Within 2 h from causative event	6/7	0/6	0/6
Vassallo [3]	2002	1	Trauma	Within 3.5 h from causative event	6/24	0/1	0/1
Katz [4]	1983	2	Trauma	Almost 4.5 h from causative event	6/12	0/2	0/2
Sun [5]	2014	8	Trauma	Almost 2.6 h from causative event	6/12	1/8	0/8
Castro [6]	2000	1	Post endoscopic sinus surgery	Almost 0.5 h from causative event	6/9	0/1	0/1
Wladis [7]	2007	1	Post endovascular procedure	Within 0.5 h from causative event	6/9	0/1	0/1
Jenkins [8]	2017	1	Trauma	48 h from causative event	nr	nr	nr
Key [9]	2008	3	Trauma	11.3 h from causative event	6/7	0/3	0/3
See [10]	2015	1	Post endoscopic sinus surgery	1 h after surgery	6/6	0/1	0/1
Colletti [11]	2017	1	Post endovascular surgery	nr	nr	nr	nr
Huang [12]	2018	1 (bilateral)	disseminated intravascular coagulation	0.5 h after onset of symptoms (epistaxis)	6/6 & 6/60	0/1	0/1
Suassez [13]	1998	2	Post endoscopic sinus surgery	1 h after surgery	6/6	0/2	0/2
Gillum [14]	1981	1	Trauma	1 h after causative event	6/7	0/1	0/1
Korinth [15]	2002	15	Trauma	70 h (2 h–15 days)	Restored in 9, defective in 4	0/15	0/15
Larsen [16]	1999	1	Trauma	2.5 h after causative events	6/7	0/1	0/1
Susarla [17]	2016	1	Post orbital floor reconstruction	14 h after surgery	6/30	0/1	0/1
Schwitkis [18]	2018	1	Trauma	1.5 h after causative events	6/6	0/1	0/1
Tran [19]	2013	1	Mastication	2 h after causative events	6/6	0/1	0/1
Sampath [20]	1995	1	Trauma	1 h after causative events	6/6	0/1	0/1
Hislop [2]	1994	2	Trauma/surgery	nr	nr	nr	nr
McInnes [21]	2002	1	Trauma	nr	nr	nr	nr
Carrim [22]	2007	1	Trauma	1.5 h after causative events	6/6	0/1	0/1
Jamal [23]	2009	1	Trauma	48 h after causative events	6/15	0/1	0/1
Maurer [24]	2013	6	Trauma	1.5 h after symptom onset	Normal vision 2/6, impaired vision 2/6, loss of vision 2/6	2/6	0/6
Pamucku [25]	2015	1	Trauma	1.5 h after symptom onset	6/7	0/1	0/1
Li [26]	1995	1	Orthognathic surgery	7 h after surgery	-	1/1	0/1
Yang [27]	2018	1	Neoplastic hemorrhage	nr	-	1/1	0/1
Amorin-Correa [28]	2017	1	Ophthalmic artery occlusion (post spine surgery prone position)	26 h after spine surgery	-	1/1	0/1
Voss [29]	2016	14	Trauma	nr	nr	3/14	0/14
Lee [30]	2006	1	Trauma	6 h after causative event	6/12	0/1	0/1
Popat [31]	2005	1	Trauma	5 h after causative event	-	1/1	0/1
Amagasaki [32]	1998	1	Trauma	“Immediate decompression”	6/6	0/1	0/1
Gauden [33]	2012	1	Intracranial surgery	nr	nr	0/1	0/1
Pahl [34]	2018	1	Intracranial surgery	nr	6/60	0/1	0/1
Yu [35]	2008	1	Spine surgery (prone position)	28 h after surgery	nr	1/1	0/1
Colletti [36]	2012	8	Trauma (2) and maxilla-facial surgery (6)	a. Almost 2.6 h from traumatic event b. 12.7 h after surgery	a. two restored vision (traumatic) b. Three impaired vision and two restored vision	a. 0/2 traumatic b. 1/6 after surgery	0/8

**Table 2:** Summary of studies about decompressive craniotomy timing

Disease	Author	Year	Number of patients	Study design	Timing of decompression	Conclusions
**Traumatic brain injury**	Shackelford [37]	2018	213	Retrospective (combat setting)	0.5–2.5 h (43 pts) 2.6–3.5 h (42 pts) 3.5–5.3 h (43 pts) 5.4–10.7 h (42 pts) 11.0 h–2.7 days (43 pts)	Postoperative mortality was significantly lower when craniectomy (DC) was initiated within 5.3 h from combat TBI.
	Barthélemy [38]	2016	12 studies 1399 patients	Systematic review		DC is of benefit (GOS) when performed < 5 h after injury in younger patients with GCS > 5.
**Acute ischemic stroke**	Dasenbrock [39]	2017	1301	Retrospective	Before 48 h (726 pts) After 48 h (575 pts)	Early decompressive craniectomy (< 48 h) was associated with superior functional outcomes. However, performing decompression before herniation may be the most important temporal consideration.
**Subarachnoid hemorrhage**	Jabbarli [40]	2017	245	Retrospective	Primary DC: 171 pts Within 24 h (120 pts) After 24 h (51 pts) Secondary DC: 74 pts	Early performance of DC (within 24 h after ictus) significantly improves the functional outcome (mRS at 6 months).
**Middle cerebral artery infarction**	Schwab [1]	1998	118	Prospective	Within 24 h (31 pts) After 24 h (32 pts) Medical Management (55 pts)	Earlier DC was associated with lower mortality. There was a trend toward better functional outcomes, and the patients spent less time in the ICU.
	Elsawaf [41]	2018	46	Prospective	DC based on deterioration of neurological status (27 pts) Within 6 h (19 pts)	Early prophylactic DC yields better clinical and radiographic outcomes than DC based on clinical status.
	Cho [42]	2003	52	Retrospective	Within 6 h (12 pts) After 6 h (30 pts) Medical management (10 pts)	DC before neurologic compromise may reduce the mortality rate and increase the conscious recovery rate.
	Mori [43]	2004	71	Retrospective	DC before herniation (21 pts) DC after herniation (29 pts) Medical management (21 pts)	Early DC before the onset of brain herniation should be performed to improve mortality and functional recovery. DC after signs of herniation may be too late for functional benefit.
	Wang [44]	2006	62	Retrospective	Within 24 h (11 pts) After 24 h (10 pts) Medical management (41 pts)	While the mortality rates were comparable between groups, severe disability may be reduced in early treated patients.
	Goedemans [45]	2020	66	Retrospective	Before 48 h (43 pts) After 48 h (23 pts)	The outcome (GOS 1-3 at 1 year) of DC performed after 48 h from stroke diagnosis in patients with malignant MCA infarct was not worse than the outcome of DC performed within 48 h.
	Lu [46]	2014	14 studies 747 patients	Meta-analysis		DC undertaken within 48 h reduced mortality and increased the number of patients with a favorable outcome (mRS) in patients with malignant MCA infarction.

*MCA* Middle cerebral artery, *AIS* Acute ischemic stroke, *GOS* Glasgow outcome scale, *DC* Decompressive craniectomy, *TBI* Traumatic brain injury, *SAH* Subarachnoid hemorrhage, *mRS* Modified Rankin scale, *GCS* Glasgow coma scale

**Table 3:** Summary of studies about decompressive laparotomy timing in abdominal compartment syndrome

Disease	Author	Year	Number of patients	Study design	Timing	Morbidity	Mortality
**Pancreatitis**	Mentula [47]	2010	26	Retrospective	DL within 4 days vs. later	nr	18% vs. 100%
	Davis [48]	2013	45	Retrospective	16 pts DL after 3.1 h from ACS diagnosis 3.3 h in BMI > 30 vs. 2.8 h in BMI < 30	43% (ECF or EAF)	Overall 24.1% 10% vs. 33.3%
	De Waele [49]	2016	33 (27 pts with primary ACS)	Retrospective	DL within 3.1 h from ACS diagnosis	24%	At 28 days: 36% At 1 year: 55%
**Burn**	Ramirez [50]	2018	46 (27 pts with ACS during initial resuscitation)	Retrospective	DL within 13 h from ACS diagnosis vs. later (analysis of the 27 pts with ACS during initial resuscitation)	nr	30% vs. 67% (*p* = 0.03)

*DL* Decompressive laparotomy, *ACS* Abdominal compartment syndrome, *pts* Patients, *ECF* Entero-cutaneous fistula, *EAF* Entero-atmospheric fistula, *BMI* Body mass index, *nr* Not reported

**Table 4:** Decompression timing

	Body district	Risk	Treatment	Clinical presentation
**Immediate**	**Eye**	Sight-threatening	Lateral canthotomy and cantholysis	Eye pain, visual loss, diplopia and reduced mobility of the eyeball. At ophthalmologic examination: eyebrow proptosis, eyelid ecchymosis, ophthalmoplegia, papilledema, and pulsation of the central retinal artery
	**Thorax** Tension pneumothorax	Cardiac arrest	Decompression: - Chest tube thoracostomy - Lateral (mini)-thoracotomy - Needle decompression	Chest pain, dyspnea, respiratory distress, tachypnea, hypoxia and/or increased oxygen requirements, increased respiratory effort and contralateral respiratory excursions, tachycardia Hyper-tympanic sound and reduction or abolition of respiratory sounds in the affected side. Absence of pleural gliding at ultrasound in the affected side.
	**Mediastinum** Cardiac tamponade	Cardiac arrest	Pericardial opening and evacuation: - Needle pericardial evacuation - Sub-xiphoidal pericardial window - Left-side thoracotomy - Clam-shell thoracotomy	Low arterial blood pressure, distended neck veins, and distant, muffled heart sounds, hemodynamic instability, shortness of breath. Pericardial free fluid at ultrasound.
**Early (within 3–12 h from diagnosis)**	**Extremities** (ECS)	Muscles necrosis	Fasciotomy	6 p’s: pain, pallor, poikilothermia, paresthesia, paralysis, and pulselessness. Pain: generally, out of proportion and exacerbated by passive stretching of the involved muscles.
	**Abdomen** (ACS^a^)	Multiorgan dysfunction syndrome	Decompressive laparotomy within 3/6 h from the diagnosis if step-up maximal medical management failed (separate considerations for severe acute pancreatitis and after burns). ^b^	Intra-abdominal hypertension with a new onset organ dysfunction.
	**Brain** (refractory elevated ICP)	Brain herniation	Decompressive craniotomy. Better outcomes in subgroups of younger patients, decompress before clinical signs of herniation.	
**Delayed (after 12 h from diagnosis)**	**Extremities** (ECS)		Fasciotomy. Discouraged for ECS occurred from > 24 h, better outcomes with non-operative management.	
	**Brain** (refractory elevated ICP)		Decompressive craniotomy. No advantage after signs of herniation in stroke patients, some advantage in traumatic brain injury even if herniated over non-surgical management. ^c^	
**Prophylactic**	**Brain** *Primary decompressive craniotomy* No ICP driven, generally utilized in TBI (also in ischemic and hemorrhagic strokes) and associated, in the acute phase, with the removal of post-traumatic intracranial hematomas	Brain herniation (presenting at the end of surgical intervention or to prevent it)		At the end of surgical intervention: - Swollen brain (impossible to reposition the bone) - Suspicion of brain swelling in next h
	**Thorax-mediastinum** (after cardiac surgery)		Open chest management. Inability for the patient to tolerate closure of the sternum after the intervention.	
	**Extremities** - Artero-venous vascular injuries - Revascularized acute limb ischemia		Prophylactic early fasciotomy (at index operation) leads to better outcomes	

^a^Abdominal pressure > 20 *plus* signs of organ failure

^b^Consider decompressive laparotomy within 1 h for ACS developed after burn injury secondary to aggressive resuscitation

^c^All benefit was lost in decompressive craniotomy performed after 48 h (Hamlet trial)

No benefit was seen in trials while the median time of decompression was 38 h

### Orbital compartment syndrome

Orbital compartment syndrome (OCS) is identified as one of the most feared ophthalmic emergencies [51]. It is a sight-threatening condition due to optic nerve and retinal compromise secondary to ischemic process deriving from a rapid and uncompensated increase of infraorbital pressure. Trauma and retrobulbar hemorrhage are the most common causes of OCS along with massive resuscitation after burn injuries. OCS is generally associated to trauma (45%) and surgery (32%) [51]. Only small increases in orbital volume can compensate thanks to the anterior globe movement and fat prolapse. Normal intraocular pressure is 3–6 mmHg (0.4–0.8 kPa). Orbital content volume is around 30 mL, including the eyeball, nerves, vessels, lacrimal glands, fat, and muscles. Orbital space and content are not fully confined and follow the pressure-volume dynamics with a pathophysiology comparable to the other compartment syndromes of the body [51, 52]. Visual loss can occur after only 60–100 min of increased pressure within the orbit [53]. Clinical judgment is of foremost importance and it should lead to the decision to decompress ocular compartment by lateral canthotomy and inferior cantholysis as soon as suggestive clinical signs (proptosis, ocular pain, loss of sight, lateral gaze limitation, evident hematoma formation) appears in the setting of a suspect for OCS [3, 4, 30].

No dedicated laboratory exams are necessary in these patients. Imaging (i.e., CT-scan of magnetic resonance-RMN) may help in defining the cause of the OCS and in differential diagnosis but they are limited to the stable patients and in case of RMN it is usually not viable in emergency setting. It is to be pointed out that imaging is to be considered complimentary and not mandatory in case of patients not amenable to be transferred especially in those with high suspicion of OCS being treated for other severe and life-threatening lesions. However, it has been demonstrated the association between a posterior globe angle of fewer than 120° and acute proptosis on CT-scan with a poorer prognosis and a higher risk of permanent vision loss [54].

Whenever OCS is suspected and clinically highly probable, the specialist should be convoked, and immediate treatment should be provided by an experienced physician. Proptosis, intraocular pressure > 40 mmHg (> 5 kPa) (whenever measured), bradycardia, and patients with lowering of the level of consciousness are indications to proceed with orbital decompression. In literature, only case reports and few case series about OCS exist. However delayed surgical intervention appears to be the most important factor affecting the rate of visual loss due to OCS [52] with a high rate of fully recovered vision in patients who are early decompressed. In general, patients treated before the 2 h from the symptoms start achieve a final Snellen visual acuity of 6/12 or better, almost 15% reach a final outcome of less than 6/12 [2, 5–27]. Outcomes in patients treated after more than 2 h are worst and almost 25% of them reach final visual acuity of 6/12 or better [5, 15, 20, 23, 24, 28, 29, 31–36, 55]. In those cases, who reached a visual acuity of 6/12 or better even if treated after 2 h, it should be clarified that more than 50% of them presented a visual acuity of 6/12 or better at the admission. Lastly, recovery of vision may be not immediate and require up to 4 weeks to reach the best outcome possible.

### Intracranial pressure and compartment syndrome of the brain

Within all compartments, the brain is reasonably the one that less tolerates a rise in pressure. Since the skull is non-expansible, a rise in brain tissue volume (e.g., edema) or blood content (e.g., hematoma) can rapidly increase the intracranial pressure (ICP) leading to a compartment syndrome that, if untreated, could bring to brain herniation and death [56, 57].

Generally, in traumatic brain injury (TBI) setting, a stepwise approach to intracranial hypertension is suggested [58]. This strategy consists of a step by step increase in the level of therapy in patients with elevated ICP reserving more aggressive interventions, generally associated with greater risks/adverse effects, when no response is observed [58]. Decompressive craniectomy (DC) is a neurosurgical procedure consisting in the removal of a part of the skull and opening the dura mater (generally by duraplasty) [59]. In this way, the increase in cranial volume allows to accommodate brain swelling because the skull is converted from a closed box (with finite volume) to an open box [59]. DC can be performed after evacuation of an intracranial lesion in the acute phase (primary, not ICP driven) or delayed (secondary) to control ICP that is refractory to maximal medical therapy [59]. DC is very effective in the ICP reduction but is considered and “extreme” therapy (being associated with several complications such as central nervous system and wound infections, cerebral hematomas, hydrocephalus) to be reserved for selected patients with refractory intracranial hypertension (IH) [58, 60]. Recent trials in TBI patients have shown differences in neurological outcome. In the DECRA trial [61], bifrontal secondary DC decreased ICP and ICU stay but was associated with more unfavorable outcomes in patients with diffuse brain injury. In the RESCUE-ICP trial [62], secondary DC (mainly unilateral) resulted in lower mortality but higher rates of vegetative state, lower severe disability, and upper severe disability. A better profile was observed in patients aged ≤ 40 years. Secondary DC was associated with a reduction in mortality in TBI but the effects on long-term neurological outcome remain controversial [63]. In this regard, future studies should focus in identifying the patients who can benefit most from this procedure considering also the most appropriate surgical techniques as well as the best timing [63]. In this regard, some studies (Table 2) suggest a benefit of early DC in terms of mortality and functional outcome [37, 38]. Literature focusing on timing of decompression however is scarce and of low quality. Even if early decompression seems to improve results, no definitive indication can be obtained from the existing trials.

DC seemed to be of benefit (decreased mortality and increased Glasgow Outcome Scale) when performed within 5 h after injury in younger patients (≤ 50 years) with a Glasgow coma scale score > 5 [38]. A retrospective analysis of 213 severe combat-related TBI undergoing DC showed a lower postoperative mortality when DC was initiated within 5.33 h from injury [37]. DC is also utilized in the management of patients with ischemic and hemorrhagic stroke [39, 46, 64]; even in this setting, the timing of DC is a matter of debate (Table 2).

The effects of DC on malignant middle cerebral artery (MCA) infarction in relation to the timing and the age of the patient has not been completely defined. Several studies and meta-analysis showed as DC undertaken within 48 h from stroke, reduced mortality, and increased the number of patients with a favorable functional outcome [39, 64]. Even analyzing narrower timeframes as before or after 6 h data confirmed the necessity to proceed with early DC before the neurological compromise arises [65]. A single-center retrospective study (66 patient) investigating the association between the timing of DC and the neurological outcome in patients with space-occupying MCA infarction showed as the 48 h cut-off in performing DC did not influence the outcome [46]. Regarding subarachnoid hemorrhage (SAH), as ingle retrospective analysis of 245 patients (171 primary DC and 74 secondary DC) focused on the value of DC timing [40]. It showed that early DC (within 24 h after ictus) significantly improves the functional outcome of SAH patients. In TBI and stroke patients, early decompression (respectively, within 6 and 24 h from injury or at least before herniation in stroke) seems to be associated with better neurological outcome. However, more data deriving from well powered clinical trial are necessary to define the appropriate timing of DC in these settings.

### Thoracic and mediastinal compartment syndrome

Different compartmental syndromes can occur within the thorax and most of them are immediately life-threatening if left untreated. Most of them are related to elective cardiac surgery. The “tight mediastinum” was described for the first time in 1975 [42].

Thoracic and mediastinal compartment syndrome has been described and open chest management has been recognized as a viable option in unstable patients who may not tolerate the chest wall closure due to an increase of intra-thoracic pressure that precipitate hemodynamic collapse at the attempt [45, 66]. During thoracic wall closure, the rise in peak inspiratory pressure may be considered an early warning for thoracic compartment syndrome [67]. Thoracic decompression must be immediate followed by open chest management with delayed sternal closure. Sternum and subcutaneous layer should be left open after surgery and mediastinal cavity should be covered with protective devices interposed between the two skin edges or the skin may eventually be temporarily sutured [67, 68]. Open chest management should be protracted until the hemodynamic conditions require it. Generally, the reported average time ranges from 2 to 7 days [69]. Decision to close may follow increasing in cardiac output, decreasing in filling pressure and improving in lung function [45]. Forcing diuresis is generally suggested; however, it should be pointed out that it may generate difficult management in patients suffering from traumatic shock.

In trauma setting, although not being officially recognized as compartmental syndromes, tension pneumothorax and cardiac tamponade both share similar pathophysiology as other component syndromes such as abdominal, in term of the rise of pressure within a space that in normal situation bears different forces. Tension PNX and cardiac tamponade must be treated by immediate decompression. Generally, there is not enough time to obtain a radiological definitive diagnosis and operative decisions must be taken on clinical conditions and suspicion [70]. E-FAST (extended focus assessment with sonography for trauma) [71] eventually is generally fundamental in corroborating the clinical suspicion. Emergency treatments range from needle decompression, to intercostal drainage, to sub-xiphoidal window, to lateral thoracotomy up to clam-shell thoracotomy.

### Abdominal compartment syndrome

Abdominal compartment syndrome (ACS) is defined as increased intra-abdominal pressure (IAP) > 20 mmHg in association with new onset organ failure; it may result in multiorgan dysfunction (i.e., cardiovascular, respiratory, renal, splanchnic, musculoskeletal, and central nervous systems) [72–75]. Due to ACS high morbidity and mortality rates, the identification of patients at risk, early recognition, appropriate staging, and timely intervention are fundamental [72].

Intra-abdominal hypertension (IAH) and ACS management must always be step-up. All medical intensive treatment must be posed into practice before proceeding to surgical decompression [76].

Clinical environment plays a pivotal role in early diagnosis and subsequent early treatment. In fact, surgical wards and surgical intensive care units (SICU) are more familiar and better trained in recognizing and timely treating IAH and ACS. Early ACS recognition and treatment increase survival rate up to 33.6% [77]. A recent study compared patients with traumatic ACS managed medical ICU (MICU) and in SICU. Median time from admission to suspicion of ACS was 60 h in the MICU vs. 13 h in SICU. After the diagnosis was done, mean time to surgery was similar in the two groups (60 vs. 53 min, respectively). Mortality was 83% in MICU and 12.5% in SICU [78]. Once diagnosed as ACS, if medical and eventual percutaneous treatments fail, it must be treated as soon as possible with decompressive laparotomy [9, 72].

De Waele et al. conducted a prospective study on 33 patients undergoing decompressive laparotomy for ACS [49, 79]. With an average time from diagnosis to decompressive laparotomy of 3 h, the overall observed 28-day mortality was of 36%, and 1-year mortality of 55%. Literature reported mortality in similar cohorts of patients was up to 50% in the first month [79]. The lowering in mortality observed in this cohort may be due to the earlier decision to proceed to decompressive laparotomy.

Specific consideration should be dedicated to IAH and ACS due to severe acute pancreatitis. In fact, indications to surgical decompression are still not clearly defined. Severe acute pancreatitis is a disease that should be treated as much as possible with intensive medical care. A step-up approach should always be adopted during the first hours from the diagnosis [80]. If no reversal was achieved with intensive medical treatment and minimally invasive attempts, proceeding to decompressive laparotomy without further waiting is mandatory [47, 75, 81]. Generally, in not improving patients with ACS, the cut-off time frame to proceed to early decompression with improved results is ≤ 6 h within the diagnosis [81]. Mentula et al. showed that early decompression (first 4 days) was associated with significantly less deaths, compared with late decompression (after 4 days) [47].

Decompressive laparotomy in severe acute pancreatitis, whenever indicated may be achieved with laparotomy (midline or transverse subcostal) or through a less invasive subcutaneous linea alba fasciotomy [82]. Some patients for sure may benefit from it (Table 3). However, despite its effectiveness in decreasing IAP and the improvement in physiological variables, no definitive data exist about the effects of surgical decompression on organ function and outcomes. Laparotomy and subsequent open abdomen management in fact are associated to significant morbidity. Davis et al. reported a comparable hospital mortality among patients with severe acute pancreatitis treated with and without decompressive laparotomy [48].

No definitive data exist on humans and some trials on animal may help in defining the effects of decompressive laparotomy (DL). Some animal models showed clearly the positive effect of early DL on metabolic derangements. A porcine model evaluating the timing of DL for ACS in severe acute pancreatitis compared four groups and found an increased survival rate in the 6 h group who underwent DL earlier (after 6 h). It was associated with a return of the normal value of urine output, blood oxygenation, and lactate clearance [83]. In another porcine model, early DL resulted in improved intestinal blood flow and could normalize the lactate/pyruvate ratio, a marker of intestinal hypoperfusion [84].

In secondary ACS, due to a reason which is not primarily abdomino-pelvic (i.e., over resuscitation), the timing of the decompressive laparotomy should be different since the pathophysiology is different as well. In patients suffering for severe burns, decompressive laparotomy may be beneficial in order to revert the effects of compartment syndrome [50, 85] (Table 3).

Ramirez et al. retrospectively analyzed a group of 46 patients with burn injury and ACS to evaluate survival comparing the timing of decompressive laparotomy [50]. Three groups were created dividing patients according to the cause of ACS development: (1) initial injury resuscitation, (2) perioperative resuscitation, and (3) sepsis. 45 patients over 46 underwent immediate laparotomy (within 1 h from ACS diagnosis) with overall survival of 56%; the age and total body surface area (TBSA) of the burn was similar in survivors and non-survivor groups. In patients with ACS associated to inhalation injury, higher mortality was observed (61% vs. 39%). The group with the highest survival rate (up to 80%) was the one of immediate DL after ACS due to initial injury resuscitation and the difference in survival was not explained by TBSA since the population with better outcome tended to have more frequently a bigger burn size and therefore more aggressively resuscitated. The authors noted increased survival rate in their population in respect of other series on ACS for a burn injury in which DL was used as the last resource, therefore concluded with the suggestion of immediate DL in ACS patients with burn injuries. It has to be stressed however that although after DL hemodynamic parameters rapidly improve, acute lung injury, and multiorgan dysfunction syndrome may be more severe after DL and more severe than in similar severely burned patients without IAH [86]. Moreover, DL is associated with higher mortality in patients aged 80 years or older. Whenever possible, DL should be avoided in frail patients [87].

### Extremities compartment syndrome

Acute extremity compartment syndrome (ECS) is due to the raise of pressure within a closed fascial compartment, causing local tissue hypoxia, and at the last stage, ischemia. ECS most often develops in the context of severe trauma, especially—nearly 75% of all cases—in long bone fractures, such as tibial and forearm fractures and crush injuries. However, it may also develop from both minor traumas, when treated with tight bandage or constrictive cast, or in non-traumatic cases. Non-traumatic ECS can develop after prolonged immobilization in unconscious patients, because of positional imbalance of the limb due to incorrect patient positioning during anesthesia in postoperative sedation [88–90], after intramuscular drug abuse or as a consequence of thermal injuries. In particular full-thickness burns may trigger ECS, causing edema and large fluid shifts in extravascular space; furthermore, the eschar may enhance the constrictive effect. Even in open fractures and open injuries, such as extremities penetrating trauma, ECS should always be ruled out. Lastly, young and fit patients seem to be more prone to develop post-traumatic ECS [91]. The diagnosis is generally clinical, and no definitive sensible and specific tools exist to undoubtedly recognize or rule out ECS. Historically, the cornerstones of clinical diagnosis were the 6 p’s: pain, pallor, poikilothermia, paresthesia, paralysis, and pulselessness. But in ECS, generally, pulses are present; if absent, other causative events should be ruled out (i.e., systemic hypotension, arterial occlusion, or vascular injury). Furthermore, the other signs are typical of delayed, missed ECS; so, for an early diagnosis, particular attention has to be paid to the pain, that is generally out of proportion and exacerbated by passive stretching of the muscles involved.

ECS early diagnosis and treatment are fundamental to avoid subsequent severe disability.

In general, longer period of ECS-related ischemia correlates with worst outcomes. Data regarding the possibility of muscles to tolerate ischemia derive from tourniquet model. These showed as muscles can tolerate up to 6–8 h of ischemia before necrosis occurs. Compartment syndrome pathophysiology in crush injury or long bone fractures setting; however, may elicit different cellular response compared to tourniquet-induced ischemia. This may result in a shorter tolerance period of ischemia by muscle bellies [92]. In fact, it was noted that victims of trauma could develop ECS even more quickly and muscle necrosis may occur even faster than the theoretical limit of 6 h [93]. There is not an exactly defined time period after which irreversible muscle damage occurs [94]. A retrospective cohort analysis showed that 37 among 76 patients who timely underwent surgery for ECS had some degree of muscle necrosis; it was estimated that nearly 37% of patients may have muscle necrosis after 3 h from injury [95]. Current surgical recommendation is based on a disease model that probably underrates the real amount of muscle damage consequent to hypoxia and ischemia. Fasciotomies for compartment syndrome performed in trauma centers of the UK were reviewed and it was found an average time of 2 h from diagnosis to fasciotomy [93]. This demonstrated that very early decompression, even in a hospital dedicated to trauma care, is uneasy to obtain, generally due to logistical issues. Authors observed a major complication rate of 34%, including limb loss, but failed to demonstrate a time-effect relationship with treatment delay [93].

ECS represents a surgical emergency: time limit for fasciotomy is within 8 h from the diagnosis of acute ECS [82, 94, 96]. In presence of obvious clinical symptoms associated with a measurement of compartment pressure higher than 40 mmHg, surgical decompression should occur within an hour.

In case of late presentation or missed diagnosis of ECS, some authors suggest non-operative management, since surgical decompression can be harmful and non-counter-balanced by reasonable benefit. It has been demonstrated that fasciotomies performed later than 8 h after diagnosis of ECS were associated with a significantly higher risk of infection [97]. In these situations, case by case evaluation is mandatory.

Lower leg fasciotomies should be performed with two incisions to decompress all the four compartment of the leg. Debridement and dressing of the surgical incisions should be performed every 48 h or more frequently if indicated [98].

Revascularization after limb vascular trauma or limb acute ischemia is the two most common causes of delayed ECS. In fact, in such cases, ECS may develop up to several hours after the end of the operation. That is why, after vascular repair procedures, it is debated if ECS should be prevented (prophylactic decompression fasciotomies) or treated once it appears.

A retrospective analysis of 612 patients who faced early or delayed (< 8 h or > 8 h) prophylactic fasciotomy (after vascular repair for lower extremity arterial injury) showed as patients with early fasciotomy had a lower rate of limb amputation (8.5 vs. 24.6% *p* > 0.001). The authors concluded that there is high suggestion to perform fasciotomy at the time of vascular repair.

Kara at al. investigated the relationship between the timing of fasciotomy and outcomes after revascularization procedures for acute limb ischemia [99]. Fasciotomies were classified as prophylactic (at the time of operation of revascularization) or delayed. One hundred and thirty-eight patients with acute limb ischemia where analyzed; 42 underwent 4-compartment fasciotomy; those who underwent delayed fasciotomy resulted in higher amputations rate within 30 days (50% vs. 5.9%, *p* = 0.002). Patients who underwent prophylactic fasciotomy had a higher Rutherford classification score.

No definitive indication can be given in regard to the prophylactic fasciotomy in acute limb ischemia. It may be performed at the time of revascularization if there is suspect of ischemia lasting for more than 6 h or of inadequate collateral flow or in the setting of trauma with a combined arterial and venous injury [100–103]. However, fasciotomy is not a riskless procedure so, in a well pondered risk-benefit balance, it may be possible to perform it only whenever ECS arises. However, it should be taken into consideration that the amputation rate with the latter approach may be higher.

### Timing of intervention

With the currently available evidence, a stratification of timing for surgical decompression has been proposed for the different body areas (Table 4). Four timing categories have been defined:

1. *Immediate decompression* for those compartmental syndromes whose missed therapy would rapidly lead to patient death or extreme disability
2. *Early decompression* with the time burden of 3–12 h and in any case before clinical signs of irreversible deterioration
3. *Delayed decompression* identified with decompression performed after 12 h or after signs of clinical deterioration has occurred
4. *Prophylactic decompression* in those situations where high incidence of compartment syndrome is expected after a specific causative event

## Conclusions

Different compartments respond with a variable degree of cellular damage and physiological decline to increased pressure; consequently, not all compartment syndromes would find benefit in timely treatment at the same extent. Although early decompression could be beneficial in some conditions, physicians must be aware of the potential harm of decompression and relative procedural hazards, thus aiming to maximize the medical effort to reduce the rate of intervention when feasible, without hesitating surgical intervention lengthy till there is no longer room for bettering outcomes. Better evidence is needed to further assess the impact of timely surgical intervention in ameliorating outcomes of patients suffering from compartmental syndromes; RTCs tailored on different body regions and timing of intervention are lacking. Given the impact of the compartment syndrome on morbi-mortality, more studies are necessary.

## Data Availability

Not applicable

## Abbreviations

ACS: Abdominal compartment syndrome
OCS: Orbital compartment syndrome
ECS: Extremities compartmental syndrome
DL: Decompressive laparotomy
TBSA: Total body surface area
mRS: Modified Rankin Score
ICP: Intracranial pressure
DC: Decompressive craniectomy
TBI: Traumatic brain injury
CS: Compartment syndrome
E-FAST: Extended focus assessment with sonography for trauma
